# Genomic prediction and genomic heritability of grain yield and its related traits in a safflower genebank collection

**DOI:** 10.1002/tpg2.20064

**Published:** 2020-11-02

**Authors:** Huanhuan Zhao, Yongjun Li, Joanna Petkowski, Surya Kant, Matthew J. Hayden, Hans D. Daetwyler

**Affiliations:** ^1^ School of Applied Systems Biology La Trobe University Bundoora VIC 3083 Australia; ^2^ Agriculture Victoria Grains Innovation Park Horsham VIC 3400 Australia; ^3^ Agriculture Victoria AgriBio, Centre for AgriBioscience Bundoora VIC 3083 Australia; ^4^ Centre for Agricultural Innovation The University of Melbourne Melbourne VIC Australia

## Abstract

Safflower, a minor oilseed crop, is gaining increased attention for food and industrial uses. Safflower genebank collections are an important genetic resource for crop enhancement and future breeding programs. In this study, we investigated the population structure of a safflower collection sourced from the Australian Grain Genebank and assessed the potential of genomic prediction (GP) to evaluate grain yield and related traits using single and multi‐site models. Prediction accuracies (PA) of genomic best linear unbiased prediction (GBLUP) from single site models ranged from 0.21 to 0.86 for all traits examined and were consistent with estimated genomic heritability (*h* ^2^), which varied from low to moderate across traits. We generally observed a low level of genome × environment interactions (g × E). Multi‐site g × E GBLUP models only improved PA for accessions with at least some phenotypes in the training set. We observed that relaxing quality filtering parameters for genotype‐by‐sequencing (GBS), such as missing genotype call rate, did not affect PA but upwardly biased *h* ^2^ estimation. Our results indicate that GP is feasible in safflower evaluation and is potentially a cost‐effective tool to facilitate fast introgression of desired safflower trait variation from genebank germplasm into breeding lines.

## Core Ideas

- Seven groups were revealed with SNPs markers in a genebank safflower collection.
- Diverse responses of safflower grain yield and its related traits to the local environments were observed, and g × E interaction patterns differed across traits.
- Moderate genomic heritability with medium to high genomic prediction accuracy was achieved for all the traits with linear mixed models.
- Quality control thresholds for genotyping‐by‐sequencing SNP data were found to affect genomic heritability estimates, but prediction accuracy was more robust.

## 1 INTRODUCTION

Safflower (*Carthamus tinctorius* L.) is a member of the family *Compositae* or *Asteraceae*, commonly referred to as the aster, daisy, composite, or sunflower family (Ashri, 1975; Elfadl, Reinbrecht, & Claupein, 2010). It is an annual, self‐compatible, diploid (2n = 2x = 24) crop with a cultivation history dating back to 3,000 B.C. in northern and central Syria (Marinova & Riehl, 2009). Safflower is a multi‐purpose crop that populated the old world as a dye plant and is also grown as a vegetable, cut flower, herbal medicine, animal feed, bird, and oilseed (Li & Mündel, 1996; Zohary, Hopf, & Weiss, 2012). Today, it is mostly grown as an oilseed crop across a broad geographical range, including Africa, America, Europe, and Asia (Emongor, 2010).

To date, limited genomic and genetic resources have been developed for safflower. Genetic diversity analyses have typically been performed using several types of sparse molecular markers including random amplified polymorphic DNAs (RAPDs) (Khan, von Witzke‐Ehbrecht, Maass, & Becker, 2009), amplified fragment length polymorphisms (AFLPs) (Johnson, Kisha, & Evans, 2007), inter‐simple sequence repeats (ISSRs) (Majidi & Zadhoush, 2014), sequence‐related amplified polymorphisms (SRAPs) (Mokhtari, Rahimmalek, Talebi, & Khorrami, 2013) and simple sequence repeats (SSRs) (Chapman et al., 2009; Usha Kiran et al., 2015). Five clusters were proposed with a world‐wide safflower collection by SSR markers, which included: 1) Europe; 2) Turkey‐Iran‐Iraq‐Afghanistan; 3) Israel‐Jordan‐Syria; 4) Egypt‐Ethiopia; 5) the Far East‐India‐Pakistan (Chapman, Hvala, Strever, & Burke, 2010). However, this was not fully supported by studies with different safflower collections (Bahmankar, Nabati, & Dehdari, 2017; Golkar & Mokhtari, 2018). More recently, low coverage, whole‐genome shotgun sequencing was used to identify millions of single nucleotide polymorphisms and to develop a high‐density genetic linkage map from a bi‐parental cross (Bowers, Pearl, & Burke, 2016). The latter provides a valuable genomic resource to support more detailed investigations of genetic diversity and genetic improvement.

While safflower has been grown world‐wide, breeding efforts for crop improvement have typically been modest. In Australia, safflower cultivation commenced in the 1940s. Coinciding with an expansion of safflower cultivation in the USA in the mid‐1950s, imported varieties were grown at a relatively small scale, mainly in Queensland and New South Wales. As a “strategic” crop in rotations or an “opportunity” crop to substitute winter crops, the safflower growing area fluctuated and peaked at 74,688 ha in 1979 (Armstrong, 1981; Jochinke, Wachsmann, Potter, & Norton, 2008). In 1987, backcrosses between the elite cultivar Gila and donor plants carrying resistance to leaf spot (*Alternaria carthami*) and root rot (*Macrophomina phaseolina*) gave rise to two Australian cultivars named “Sironaria” and “Sirothora”. Despite these important varieties, the safflower growing area declined dramatically in Australia, in part aided by a sudden collapse of Australian safflower crushing capacity (Harrigan, 1987). In 2000, research evaluating the potential role of safflower in southern region cropping systems (Victoria and South Australia) highlighted its potential as a “cash” crop (Mailer RJ, Redden, & Ayton, 2008; Wachsmann, Jochinke, Potter, & Norton, 2008). In recent years, the demand for healthy cooking oil, clean biofuel, and industrial lubricants has surged, and safflower, with its high oleic acid content, has gained more attention (Khalid et al., 2017; L & Ll, 2016). Breeding for high oil yielding varieties with the ability to adapt to the existing farming system in Australia is, therefore, of importance.

Genomic prediction (GP) uses genome‐wide molecular marker information with quantitative genetic techniques to predict the breeding value of individuals, which facilitates the evaluation of plant performance and selection for breeding candidates (Crossa et al., 2017; Voss‐Fels, Cooper, & Hayes, 2019). By using thousands of markers covering the whole genome, GP assumes that all causal variants are in linkage disequilibrium with at least one SNP. Theoretically, GP can account for all causal variants underlying a trait and is expected to outperform pedigree or marker‐assisted selection with a few markers for complex traits (Goddard & Hayes, 2007; Meuwissen, Hayes, & Goddard, 2001). GP utilizes a training population, which has been genotyped and phenotyped, to develop a statistical model to predict individuals that have been genotyped but not phenotyped. The predicted breeding values are known as genomic estimated breeding values (GEBV) (de los Campos, Hickey, Pong‐Wong, Daetwyler, & Calus, 2013). Predicted GEBV can help to identify the best performing individuals as potential commercial cultivars or potential donor parents for future crosses in different breeding populations (Pembleton et al., 2018; Slater, Cogan, Forster, Hayes, & Daetwyler, 2016). The integration of GP into the genebank germplasm evaluation process demonstrated that directly mining genetic diversity from genebanks for future breeding programs is feasible (Muleta, Bulli, Zhang, Chen, & Pumphrey, 2017; Yu et al., 2016). The accuracy of GP has been shown to be moderate to high in a range of crops, indicating its feasibility for genetic improvement. In a pea diversity panel, prediction accuracy (PA) ranged from 0.29 to 0.62 for flowering time, seed number, and seed weight (Burstin et al., 2015). Moderate GP accuracy was achieved for wheat leaf, stripe, and stem rust resistance (Daetwyler, Bansal, Bariana, Hayden, & Hayes, 2014). A study in maize landraces achieved accuracies ranging from −0.57 to 0.74 for seven agronomic traits within or among doubled haploid populations (Brauner et al., 2018).

**Core Ideas**

- Seven groups were revealed with SNPs markers in a genebank safflower collection.
- Diverse responses of safflower grain yield and its related traits to the local environments were observed, and g × E interaction patterns differed across traits.
- Moderate genomic heritability with medium to high genomic prediction accuracy was achieved for all the traits with linear mixed models.
- Quality control thresholds for genotyping‐by‐sequencing SNP data were found to affect genomic heritability estimates, but prediction accuracy was more robust.

The evaluation and utilization of diverse germplasm collections are essential for crop improvement (Joukhadar, Daetwyler, Bansal, Gendall, & Hayden, 2017; Lombardi et al., 2014). In this study, safflower accessions conserved in the Australian Grain Genebank (AGG) were phenotyped in four field trials and genotyped with a genotyping‐by‐sequencing (GBS) platform. We used these diverse safflower genebank accessions as the training population to study the potential of GP in safflower germplasm evaluation with the aim to 1) investigate the population structure and genetic diversity of the collection; 2) estimate genomic heritability and evaluate GP accuracies for grain yield and its related traits; and 3) explore the application of GBS data in safflower GP.

## 2 MATERIALS AND METHODS

### 2.1 Plant material and phenotyping

A total of 387 safflower accessions were sourced from the AGG with the aim of compiling a diverse reference population for genomic prediction. Accessions were bulked in the glasshouse, and two replicated field trials were sown in each 2017 and 2018 for a total of four field trials with field entries varying from 368 to 387 accessions (Supplemental Table S1).

Two safflower trials were sown in 2017 in Horsham, Victoria, Australia (36.70° S, 142.20° E). Site 1 was planted in a fallow field with flood irrigation before sowing, and Site 2 was rotated after wheat without irrigation. In 2018, one trial was sown in Horsham (low‐rainfall zone), the same location as Site 2, and referred to as Site 3, and the other trial was sown in Site 4 located in Wonwonda (a higher‐rainfall zone), Victoria, Australia (36.90° S, 142.30° E). Soil pH ranged from 7.5–9.0 for all field trials. A randomized complete block design with three replicates in 2017 and two replicates in 2018 was adopted. Plot dimensions were 1 m by 5 m with five rows in each plot, and 220 seeds per plot were sown for all four sites.

Morphological notes were recorded during the growing season. Leaf spininess was recorded at the onset of flowering as a 0 = spineless, 1 = minor spine, 2 = spine. Flower color was recorded as fresh flower color/dry flower color as o = orange, r = red, y = yellow, and w = white. Leaf margin was recorded as 1 = entire, 2 = serrate or dentate, 3 = deeply serrate, and 4 = lobed, according to safflower descriptors (IBPGR, 1983). Seed color was recorded as 1 = white, 2 = creamy, and 3 = other color.

Days to flowering (DF) was recorded as the number of days from sowing to 25% plot flowering. Flowering period (FP) was recorded as days from 25% plot flowering to 90% of the plot ending flowering. Days to maturity (DM) were recorded as days from sowing to 90% of the plot being physiologically mature. Plant height (PH) was measured at the late flowering stage from the ground surface to plant natural height in cm. After harvest, grain yield was measured as the total plot seed weight (yield per plot, YP) in kilograms, and 500 seed weight (SW) was assessed from 500 randomly sampled seeds per plot.

### 2.2 Genotyping

A total of 349 safflower accessions were genotyped. Genomic DNA was extracted from six crushed seeds per accession using an Agencourt DNAdvance genomic DNA isolation kit according to the manufacturer's instructions. Genotyping‐by‐sequencing was performed by digesting genomic DNA for each accession with the restriction endonucleases *Pst*I (6‐bp cutter) and *Mse*I (4‐bp cutter), followed by the ligation of adapters. Two types of adapters were used: one for ligation to *Pst*I cut sites, which had a core sequence suitable for PCR amplification using standard Illumina PE1 primers, and the other for ligation to *Mse*I cut sites, which had a core sequence suitable for PCR amplification using standard Illumina PE2 primers. PCR amplification of adapter‐ligated *PstI‐Mse*I genomic restriction fragments was performed using barcoded Illumina PE1 and PE2 primers and the following amplification profile: 30 cycles of 98 °C for 10 s, 65 °C for 30 s, 72 °C for 30 s. The resulting barcode indexed amplicons for each accession were pooled in equimolar proportions, purified using Agencourt AM Pure magnetic beads (Beckman Coulter) at the ratio of 1:0.8 (library: beads) and sequenced on the Illumina Hiseq 3000 sequencer. Custom scripts were used to filter the resulting sequence data and for SNP discovery and genotype calling.

### 2.3 Population structure

A total 32,642 codominant SNP markers were identified and, after filtering for missing rate < 30% and MAF > 0.01, were used to calculate the Roger's distance matrix in R (Reif, Melchinger, & Frisch, 2005). Population clustering was based on a Neighbour‐Joining dendrogram calculated on Roger's distance matrix, and an unrooted phylogenetic tree was generated with the R package “ape” (Paradis & Schliep, 2018). SNPs were imputed with LinkImpute, a software package based on a k‐nearest neighbor genotype imputation method (LD‐kNNi) for unordered markers (Money et al., 2015). A genomic relationship matrix (**G**), reflecting the genetic relatedness between accessions, was calculated from imputed SNPs with MAF > 0.05, according to VanRaden (VanRaden, 2008). Genotype SNP data for the349 safflower accessions selected for G was included in the Supplemental Table S5. Briefly, **G** was calculated as $G=\frac{\mathrm{ZZ}\prime }{2\sum p_{i}\left(1-p_{i}\right)}$, where *p_i_* is the allele frequency at allele *i*, **Z** is calculated from matrix **M** (n × m marker matrix with n individuals and m markers) with **P** (calculated as 2(p_i_‐0.5) for each allele *i*) subtracted. A heatmap sorted by hierarchical clustering (dendrogram) based on **G** was computed in R (R Core Team, 2014). Principal component analysis (PCA) with the same dataset was conducted with a R script (R Core Team, 2014).

### 2.4 Statistical analysis

#### 2.4.1 Broad‐sense heritability (*H* ^2^)

All phenotypic data collected in the field were adjusted by fitting the autoregressive (AR) linear mixed model, also known as best linear unbiased prediction (BLUP) model:

(1) $$y=\mathrm{Xb}+Z_{g}g+Z_{r}r+Z_{c}c+Z_{\mathrm{rep}}\mathrm{rep}+e$$

where **y** is a vector of phenotypic values for each accession, **b** is a vector of fixed effects including the mean, **g** is a vector of random genetic effects of accessions ∼ *N* (0, $\sigma_{G}^{2}$ **I**), **I** is an identity matrix, $\sigma_{G}^{2}$ is the genetic variance due to accessions, **r**, **c**, and **rep** are vectors of random field design effects, respectively for the row, column, and replication; **e** includes the independent error ∼*N*(0, $\sigma_{e}^{2}$ **I**), and the spatial dependent error which is the row and column two dimensional covariance, $\;\sum_{r}\left(\rho_{r}\right)⊗\sum_{c}\left(\rho_{c}\right),\;$ (Gilmour et al., 2015). **X**, **Z_g_**, **Z_r_**, **Z_c_**, and **Z_rep_** are the corresponding design matrices for **b**, **g**, **r**, **c**, and **rep**, respectively. Variance components were estimated in ASReml (Gilmour et al., 2015) with restricted maximum likelihood (REML). The phenotypic variance (V_p_) was partitioned into the genetic variance (V_G_), and the experimental design variances (V_E_, V_E_ = $\sigma_{r}^{2}$ +$\sigma_{c}^{2}$+$\sigma_{rep}^{2}$+ $\sigma_{G}^{2}$/rep), where rep was the number of the replications per accession.

Broad‐sense heritability (*H* ^2^) was calculated as the proportion of phenotypic variance (V_P_) explained by genetic variance (V_G_):

$$H^{2}=V_{G}/V_{P}=\sigma_{G}^{2}/\sigma_{P}^{2}$$

#### 2.4.2 Genomic heritability (*h* ^2^) and genomic × environment interaction

The adjusted phenotypes, also known as best linear unbiased estimates (BLUEs), were calculated from model 1 by fitting the accessions as a fixed effect (given in Supplemental Table S4). The 349 genotyped safflower accessions were chosen for the genomic heritability and g × E interaction analysis.

The genomic BLUP (GBLUP) model replaced matrix **I**, with **G**, illustrated as:

(2) y=Xb+Zg+e

where **y** is a vector of BLUEs of each accession, **X** and **Z** are design matrices, b is the mean, **g** is a vector of random additive genetic effects distributed as *N* (0, $\sigma_{A}^{2}$ **G**), $\sigma_{A}^{2}$ is the additive genetic variance captured by SNP markers, **e** is the residual with *N*(0, $\sigma_{e}^{2}$ **I**), and $\sigma_{e}^{2}$ is the residual variance. The proportion of the additive genetic variance captured by SNPs to the total variance, known as genomic heritability (*h* ^2^), for each trait at each site was calculated as:

$$h^{2}=\sigma_{A}^{2}/\left(\sigma_{A}^{2}+\sigma_{e}^{2}\right)$$

The four field sites conducted in two consecutive years were combined in an extended GBLUP (g × E GBLUP) model that included an interaction between the site and additive genetic effects:

(3) $$y=\mathrm{Xb}+\mathrm{Zg}+Z_{2}\mathrm{gE}+e$$

where **b** is a vector of fixed effects including the overall mean and site, gE is the interaction between site and additive genetic effects distributed as *N*(0, $\sigma_{AE}^{2}G⊗I$), **G** is the genomic relationship matrix, **I** is the 4 × 4 incident matrix, and ⊗ is the Kronecker or direct product. $\sigma_{AE}^{2}\;$is the variance of the interaction effect, **Z_2_** is the incidence matrix relating the BLUEs to the interaction effect, and **e** is the residual. To test the significance of the gE interaction effect, the likelihood ratio test was conducted for all the traits with a R script.

The ratio of additive genetic variance to the total variance ($\sigma_{AE}^{2}$ +$\;\sigma_{A}^{2}$ +$\;\sigma_{e}^{2}$) was defined as the multi‐site genomic heritability (*h* ^2^_ge_), which also could be defined as repeatability. With the assumption that the variation within individuals across the environments is caused by measurement errors and random environmental factors, it measures the repeatability of the additive genetic effects across the environments. It was calculated as below with *s* as the number of sites:

$$h_{ge}^{2}=\sigma_{A}^{2}/\left(\sigma_{A}^{2}+\sigma_{AE}^{2}/s+\sigma_{e}^{2}/s\right)$$

The level of g × E interaction was evaluated as the proportion of the interaction variance ($\sigma_{AE}^{2}$) to the total additive genetic variance ($\sigma_{A}^{2}+\sigma_{AE}^{2}$).

#### 2.4.3 Cross‐validation and genomic prediction accuracy

Two models were used to predict GEBVs: a GBLUP model (model 2) and a g × E GBLUP model (model 3), as described above to estimate genomic heritability, but always excluding the phenotype of the validation set. GP accuracy was evaluated with a fivefold cross‐validation method. The total accessions in each site were randomly divided into five equal subsets and each of the subsets was, in turn, chosen as the validation sample set and was subsequently predicted by using the other four subsets as the training population. Prediction accuracy (PA) was calculated as the Pearson's correlation coefficient between GEBVs and BLUEs. The cross‐validation was repeated five times, and the mean PA, standard deviation (SD), and the mean slope of the regression of BLUEs on GEBVs at each site were calculated across all 25 subsets.

In the g × E GBLUP model, two prediction scenarios were compared. The first scenario (CV1) predicted untested genotypes that have not been phenotyped at any site. PA was calculated for validation samples at each site. The second scenario (CV2) predicted the performance of the genotypes that have been evaluated in some sites but not in others, where PA was calculated for the validation sample at each of the missing sites.

#### 2.4.4 Impact of GBS data quality control on heritability and prediction accuracy

While GBS genotyping is commonly used in breeding programs, its high missing genotype call rate can be challenging for down‐stream analysis. To investigate the impact of SNP data quality control on GP and *h* ^2^ estimation, we tested different missing genotype rates, set at 10%, 30%, 50%, and 80%. The software LinkImpute was applied, and the imputation accuracy was calculated by random masking the known genotypes in the dataset (Money et al., 2015). **G** was calculated according to VanRaden (2008) as above, with two MAF cut off points (MAF > 0.01 and MAF > 0.05). LD between all pairs of SNP markers was calculated as the squared correlation (r^2^) in PLINK (Purcell et al., 2007). PA and *h* ^2^ for grain yield (YP) in four sites were estimated for each parameter combination with GBLUP model.

## 3 RESULTS

### 3.1 Genotypes and population structure

**FIGURE 1:**
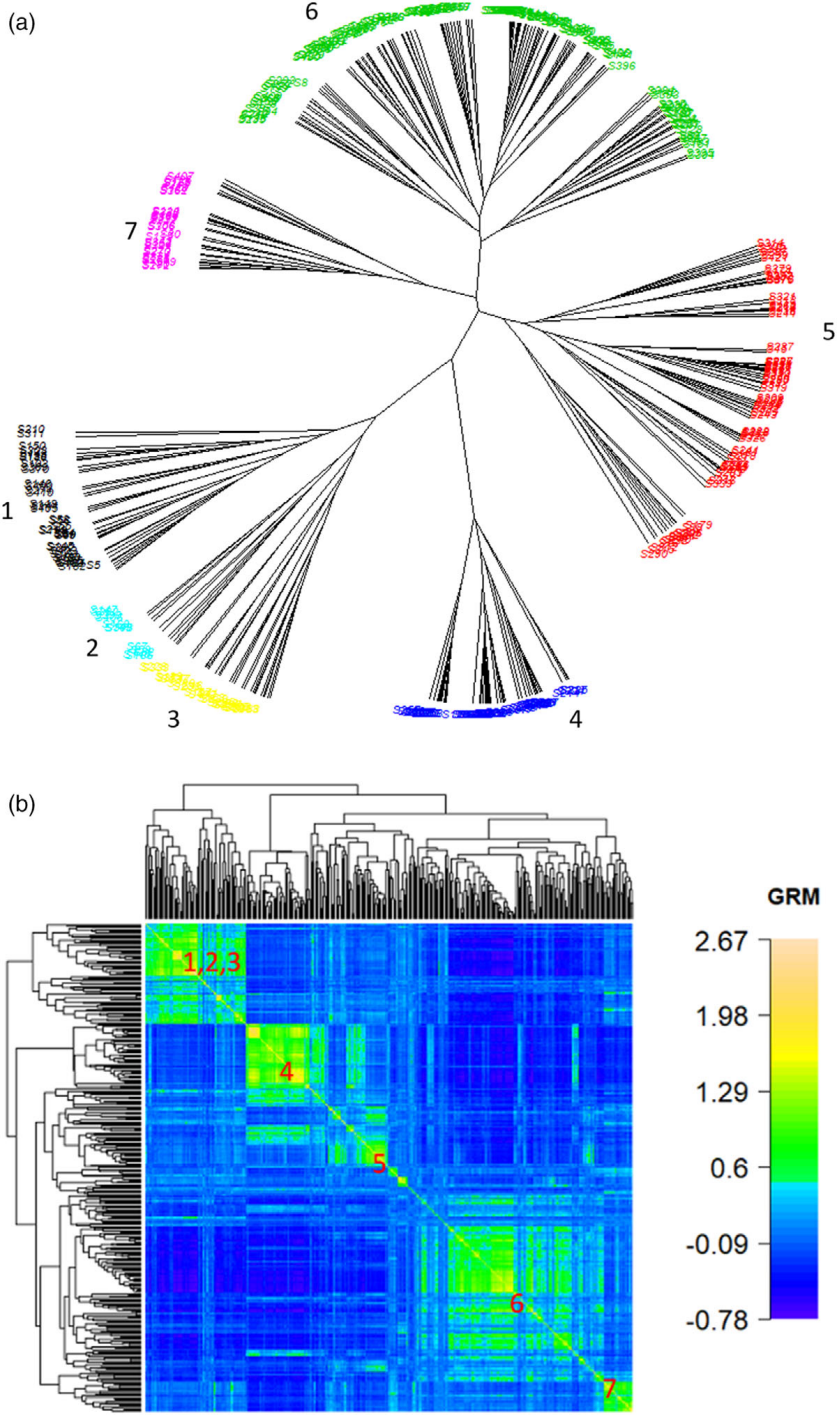
(a) Top panel. Unrooted phylogenetic tree of 349 safflower accessions with seven groups (defined by the different color codes). (b) Bottom panel. Heatmap of genomic relationships based on G among 349 safflower accessions aligned with the dendrogram. Higher values in G mean higher relatedness between accessions. The group numbers correspond to groups in Figure 1a

A total of 3,177 SNP passed quality control and were included in the population structure analysis. Seven groups were revealed by the dendrogram, and the unrooted phylogenetic tree showed that groups 1, 2, and 3 formed one branch, while groups 4, 5, 6, and 7 were more differentiated (Figure 1a). In the heatmap based on G, we also observed that groups 1, 2, and 3 were highly related to each other. Accessions from Iran mainly included in groups 1 and 3. However, no clear clustering by country of origin was observed. Morphology characteristics differed groups 1, 2, and 3 from other groups. With total 72 accessions in groups 1, 2, and 3, the main morphology characteristics are spineless leaves (58%), smooth and dentate leaf margins (89%), white achenes (67%), and late‐flowering types. Sporadic relatedness across groups 4, 5, 6, and 7 was revealed in the heatmap, but was much lower than within group relatedness. It indicated some genetic linkage was present among those groups (Figure 1b). Group 4 was dominated by landraces from India and Pakistan with spiny leaves, and accessions within group 4 are highly related. Spiny leafed landraces from Portugal, Ethiopia were grouped in group 5. However, accessions from other countries were also included in group 5. The relatedness of accessions within group 5 was less than in groups 4 and 6, and it indicated that groups 5 was more diverse. Most advanced cultivars and breeding lines were in Group 6. Landraces from Israel, Jordan, Syria mainly grouped in group 7 (Supplemental Table S2). PCA analysis showed that the first and the second principal components (PC1, PC2) explaining 51% and 24% of the total variance, respectively (Supplemental Figure S1).

### 3.2 Summary statistical analysis of 349 safflower accessions

**FIGURE 2:**
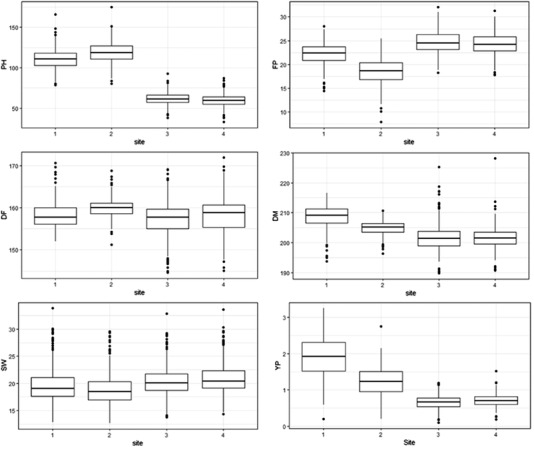
Box‐plots of 349 safflower accessions’ average and standard deviation of adjusted phenotypes for days to flowering (DF), days to maturity (DM), flowering period (FP), plant height (PH), grain yield (YP), and 500 seed weight (SW) in four sites

The safflower genebank accessions showed diverse phenotypes for all the traits studied across four sites (Supplemental Figure S2). For the 349 safflower accessions, grain yield (YP) ranged from 0.2 to 3.25 kg, with an average of 1.88 kg at Site 1. While at Site 2 the average YP was 1.22 kg, approximately 2/3 of Site 1. Sites 3 and 4 produced about half of the average YP of Site 1 (Figure 2). Plant height (PH) varied from 80.5 cm to 174.8 cm among accessions at Site 2. The average PH at Sites 3 and 4 was close to half of that observed at Site 2, indicating poor plant growth at those two sites. The average 500 seed weight did not vary appreciably between sites and ranged from 12.79 grams to 33.86 grams at Site 1. For the phenology traits, the average of days to flowering (DF), flowering period (FP), and days to maturity (DM) did not differ dramatically across the four sites. High phenotypic correlations between DF and DM were observed among sites (r = 0.4–0.7). At Site 1, the DF commenced at 152 days, with the late‐flowering lines flowering at 170 days, giving a range of 22 days with an average of 158 days. The FP ranged from 14 to 28 days, and the DM spanned from 193 to 216 days across the four sites.

### 3.3 Variance components and heritability estimation

The phenotypic variance ($\sigma_{P}^{2}$) and the total genetic variance ($\sigma_{G}^{2}$) estimated with the BLUP model differed among sites for each trait (Table 1). At Sites 3 and 4, $\sigma_{P}^{2}$ and $\sigma_{G}^{2}$ shrunk dramatically compared to Sites 1 and 2 for YP and PH. Phenology traits showed different patterns on Site 2, where both $\sigma_{P}^{2}$ and $\sigma_{G}^{2}$ decreased for DF and DM and increased for FP. The additive genetic variance ($\sigma_{A}^{2}$) captured by the SNP markers in the GBLUP model was smaller than the total genetic variance ($\sigma_{G}^{2}$) for all traits. With the combined g × E GBLUP model, the gE interaction effects were significant for all traits studied. With the gE interaction effect included, FP and SW had a higher $\sigma_{A}^{2}$ estimated by the g × E GBLUP model than the average $\sigma_{A}^{2}$ across the four sites by the GBLUP model.

**TABLE 1:** Variance components and heritability with standard error (in the parentheses) estimated from BLUP, GBLUP and g × E GBLUP models

		BLUP			GBLUP			g × E GBLUP			
Trait	Site	$\sigma_{P}^{2}$	$\sigma_{e}^{2}$	*H* ^2^	$\sigma_{A}^{2}$	$\sigma_{e}^{2}$	*h* ^2^	$\sigma_{AE}^{2}$	$\sigma_{A}^{2}$	$\sigma_{e}^{2}$	*h* ^2^_ge_
Plant height (PH)	1	241.64	205.39	0.85(0.03)	70.33	35.83	0.66(0.08)	10.99	36.26	24.15	0.80(0.03)
	2	192.35	159.65	0.83(0.02)	47.63	31.67	0.6(0.08)				
	3	61.33	30.05	0.49(0.04)	10.58	29.36	0.26(0.08)				
	4	69.51	38.23	0.55(0.06)	17.57	18.47	0.49(0.09)				
	Av.			**0.68(0.04)**			**0.50(0.08)**				
Days to flowering (DF)	1	11.53	10.49	0.91(0.02)	3.31	1.98	0.63(0.09)	1.31	4.15	1.57	0.85(0.03)
	2	10.24	4.71	0.46(0.05)	1.61	2.68	0.37(0.09)				
	3	18.96	18.39	0.97(0.01)	4.49	2.14	0.68(0.07)				
	4	19.34	18.76	0.97(0.01)	5.2	2.71	0.66(0.08)				
	Av.			**0.83(0.02)**			**0.58(0.08)**				
Flowering period (FP)	1	6.28	5.21	0.83(0.03)	1.95	2.61	0.43(0.09)	0.68	2.99	2.48	0.79(0.04)
	2	10.67	7.47	0.70(0.05)	1.73	3.04	0.36(0.08)				
	3	6.72	4.64	0.69(0.04)	1.35	4.21	0.24(0.09)				
	4	6.34	5.01	0.79(0.03)	1.44	4.39	0.25(0.09)				
	Av.			**0.75(0.03)**			**0.32(0.09)**				
Days to maturity (DM)	1	16.61	15.28	0.92(0.02)	4.14	3.35	0.55(0.08)	0.95	5.55	2.56	0.86(0.03)
	2	5.34	4.11	0.77(0.03)	1.61	1.33	0.55(0.08)				
	3	22.03	16.3	0.74(0.03)	6.88	5.68	0.55(0.08)				
	4	13.81	11.74	0.85(0.02)	6.42	3.35	0.66(0.08)				
	Av.			**0.82(0.02)**			**0.58(0.08)**				
Grain yield (YP)	1	497.45	397.96	0.80(0.03)	81.58	154.19	0.35(0.09)	16.65	39.44	57.46	0.68(0.04)
	2	231.96	122.94	0.53(0.04)	45.53	73.04	0.38(0.08)				
	3	49.43	20.76	0.42(0.04)	5.85	27.7	0.17(0.08)				
	4	40.27	18.12	0.45(0.03)	8.64	18.08	0.32(0.09)				
	Av.			**0.55(0.04)**			**0.31(0.09)**				
500 Seed weight (SW)	1	10.78	10.46	0.97(0.01)	5.34	2.61	0.67(0.08)	0.06	8.45	0.72	0.97(0.01)
	2	9.84	9.35	0.95(0.01)	5.04	1.78	0.74(0.07)				
	3	9.31	8.94	0.96(0.01)	3.66	2.2	0.62(0.08)				
	4	9.45	9.17	0.97(0.01)	3.75	2.26	0.62(0.08)				
	Av.			**0.96(0.01)**			**0.66(0.08)**				

*Note*. $\sigma_{P}^{2}$ is the phenotypic variance, $\sigma_{G}^{2}$ is the total genetic variance, and $\sigma_{A}^{2}$ is the additive genetic variance captured by the SNPs markers, $\sigma_{AE}^{2}$ is the variance for g × E interaction, and $\sigma_{e}^{2}$ is the residual variance. *H* ^2^ is the estimated broad‐sense heritability, *h* ^2^ is the genomic heritability for single site, and *h* ^2^_ge_ is the multi‐site genomic heritability with standard errors (in the parentheses). Av. is the average of the estimated heritability.

Three heritability was estimated in this study using the linear‐mixed models for all traits studied: broad‐sense heritability (*H* ^2^), (narrow‐sense) genomic heritability at single sites (*h* ^2^) and multi‐site genomic heritability (*h* ^2^_ge_) (Table 1). Accessions were treated as independent individuals for *H* ^2^ estimation in the BLUP model. While for *h* ^2^ and *h* ^2^_ge_ estimation, a **G** derived from SNP markers was used to represent the relationship between accessions. SW, DF, and DM had a high estimated *H* ^2^ above 0.80 and moderate *h* ^2^. Both *H* ^2^ and *h* ^2^ were low for YP at 0.55 and 0.31, respectively. PH had moderate *H* ^2^ (0.68) with moderate *h* ^2^ (0.50), while FP showed moderate‐high *H* ^2^ (0.75) with low *h* ^2^ (0.32). Estimated *H* ^2^ and *h* ^2^ were lower for PH and YP at Sites 3 and 4, and for DF at Site 2. *h* ^2^_ge_ were much higher than the mean *h* ^2^ across sites for all traits studied. The g × E interaction levels varied, but were low for all traits studied, with the interaction variance was 30% of the total additive genetic variance for YP, 15 to 24% for PH, DF, FP, and DM, and 0% for SW.

### 3.4 Cross‐validation and genomic prediction accuracy

Moderate to high average prediction accuracy (PA) of approximately 0.70 across the four sites was achieved for PH, DF, DM, and SW, while PA was lower at 0.47 for FP and YP with the single site GBLUP (Table 2). The PA of the g × E GBLUP CV1 scenario was comparable with the single‐site GBLUP for all the traits studied. In the CV2 scenario, g × E GBLUP resulted in higher PA than single site GBLUP for all traits. SW reached the highest average PA at 0.94 in the CV2 scenario with g × E GBLUP. The slope of the regression of BLUEs on estimated GEBVs was expected to be 1, and a deviation from 1 indicates that GEBVs were over or underestimated. The average slopes of around 1 for all traits and all GP models demonstrate that the GEBVs were unbiased (Table 2). However, at some sites, the slopes deviated considerably from 1 in the CV1 and CV2 scenarios (Supplemental Table S3).

**TABLE 2:** The mean prediction accuracy (PA) of GEBVs and their standard deviations (in parentheses), and the average slope (Avg_slope) of the regression of BLUEs on GEBVs estimated from GBLUP (GBLUP_CV) and g × E GBLUP (CV1 and CV2) for all phenotypic traits

	GBLUP_CV		g × EGBLUP_CV1		g × EGBLUP_CV2	
Trait	PA	Avg_slope	PA	Avg_slope	PA	Avg_slope
Plant height (PH)	0.70(0.06)	1.00	0.69(0.06)	0.94	0.77(0.05)	0.99
Days to flowering (DF)	0.72(0.06)	1.00	0.71(0.06)	0.94	0.81(0.04)	1.02
Flowering period (FP)	0.46(0.10)	1.05	0.48(0.08)	0.91	0.65(0.08)	1.05
Days to maturity (DM)	0.73(0.05)	1.01	0.72(0.05)	0.96	0.84(0.04)	1.06
Grain yield (YP)	0.47(0.09)	1.04	0.44(0.10)	0.98	0.58(0.08)	1.14
500 Seed weight (SW)	0.69(0.08)	0.98	0.67(0.06)	0.84	0.94(0.02)	1.00

### 3.5 Effect of GBS data quality control on genomic heritability and prediction accuracy

A total of 349 safflower accessions with 32,642 SNP were analyzed in this study. Increasing the SNP missing rate from 10% to 80% dramatically increased the number of SNP from 1,419 to 15,764 (Table 3). Imposing a MAF threshold of 0.05, instead of 0.01, approximately halved the SNP number. As the missing rate increased, relatedness in **G** within (diagonals) and across (off diagonals) individuals also increased. There was no difference of **G** (diagonals and off diagonals) between the two MAF thresholds, while higher average LD was observed at MAF cut‐off 0.05 than at 0.01. The average LD between all pairwise SNPs was similar when at 10% or 30% SNP missing rates but decreased substantially at 50% and 80% SNP missing rates.

**TABLE 3:** SNP number, imputation accuracy, mean of genomic relationship matrix diagonals (DiagG) and off‐diagonals (Off‐diagG), and average LD of pairwise SNPs (avgLD) with different SNP missing rates and minor allele frequency (MAF) thresholds

			MAF > 0.01				MAF > 0.05			
SNP missing rate	SNPs	Imputation Accuracy	SNPs	Diag**G**	Off‐diag**G**	avgLD	SNPs	Diag**G**	Off‐diag**G**	avgLD
10%	1419	0.932	1132	1.488	−0.00425	0.025	607	1.481	−0.00423	0.044
30%	3852	0.929	3221	1.546	−0.00441	0.023	1787	1.542	−0.0044	0.043
50%	6911	0.925	5931	1.571	−0.00448	0.017	3251	1.569	−0.00448	0.033
80%	15764	0.918	14427	1.622	−0.00463	0.01	7885	1.617	−0.00462	0.018

**FIGURE 3:**
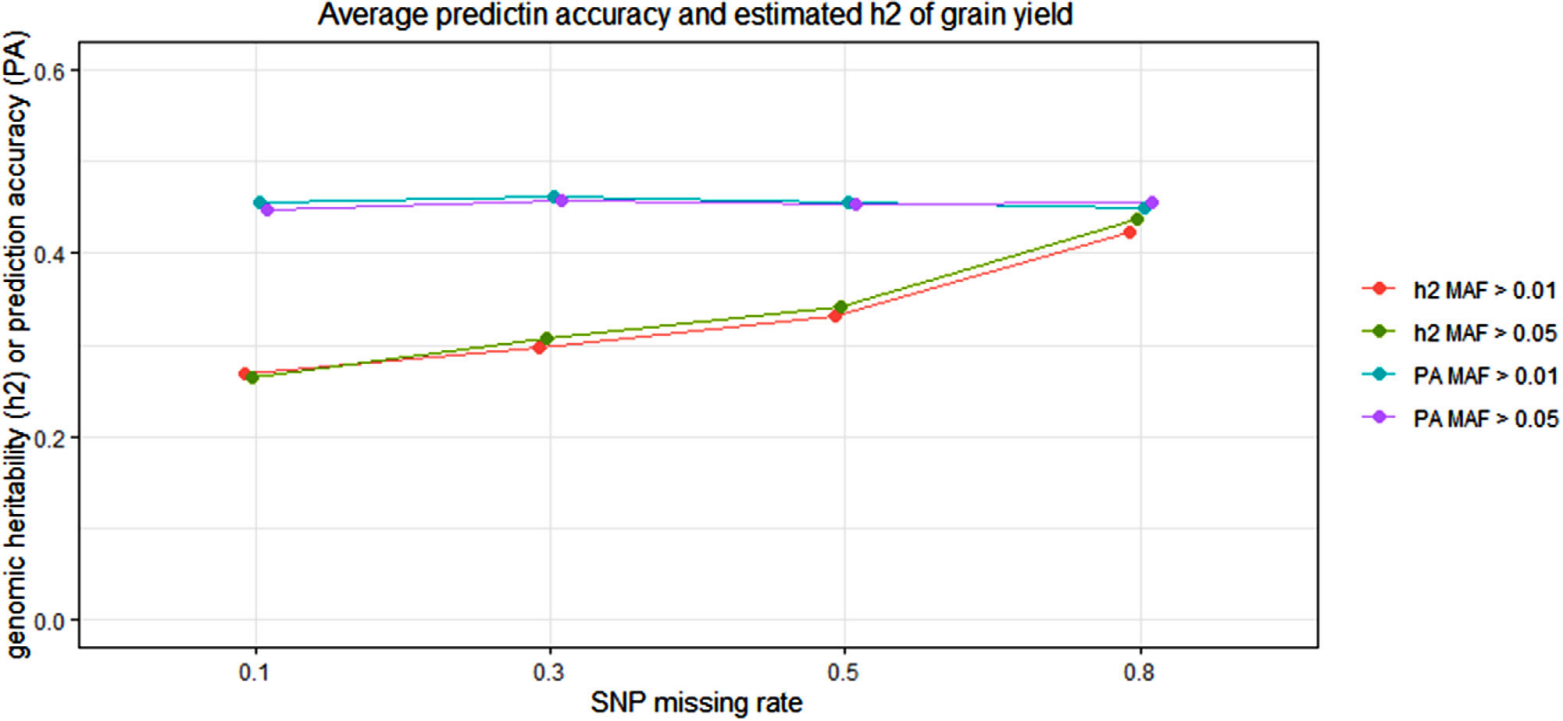
Average prediction accuracy (PA) and estimated genomic heritability (*h* ^2^) across the four sites with a combination of SNP missing rates and minor allele frequency (MAF) cut‐offs for grain yield (YP)

Grain yield was investigated as an example to examine the effect of SNP missing rates on the genomic prediction accuracy (PA) and genomic heritability estimation. In general, the PA for grain yield was constant with the SNPs from different missing rates and MAF thresholds, but the estimated *h* ^2^ tended to increase as SNP missing rates increased. This was most dramatic at 80% SNP missing rate for both MAF thresholds (Figure 3).

## 4 DISCUSSION

Although safflower is grown world‐wide, as a minor crop, limited genomic and genetic resources have been developed (Golkar & Mokhtari, 2018). The genome‐wide SNPs generated in our study with the GBS platform are important genetic resources for safflower research. With more than 3000 genome‐wide SNPs, we revealed that the AGG safflower collection could be divided into seven main groups. Iranian accessions tended to cluster within groups 1 and 3, differed from other groups, indicating their unique genetic background compared to accessions from other countries. This finding is consistent with recent studies using collections from Iran–Afghanistan regions (Bahmankar et al., 2017; Kumar et al., 2015). The accessions from Europe, Israel‐Jordan, and India‐Pakistan clustered into the different groups in our study were consistent with studies using molecular markers other than SNPs (Chapman et al., 2010; Golkar & Mokhtari, 2018). It suggested that accessions from the traditional safflower growing area shared more genetic similarity within the area, and very limited germplasm exchange happened between these areas. High genetic diversity has been reported previously within India‐Pakistan safflower accessions (Kumar et al., 2015). However, the accessions from India‐Pakistan in our study showed narrow genetic diversity, and this could be due to the limited number of accessions collected from the India‐Pakistan region in the AGG. Interestingly, accessions from the same country of origin were sometimes spread across different subgroups. For example, accessions from Turkey were observed in every group. The potentially admixed genetic background of Turkey accessions has also been observed previously (Ali et al., 2019; Lee et al., 2014). This admixture could arise from the geographic position of Turkey, linking Europe and Asia, which facilitated the frequent germplasm interchange. All elite cultivars and breeding lines in the AGG collection grouped into one group, indicating their narrow genetic base, which supports the suggestion that most safflower breeding lines originate from a single gene‐pool (Pearl & Burke, 2014). The diverse population structure revealed in our study showed that the safflower collection housed in the Australian Grains Genebank is a valuable genetic resource for future safflower breeding.

Safflower is grown widely in dry and semi‐dry areas. The Wimmera region of western Victoria is a potential safflower growing area in Australia, and the field trials mainly focussed on this region without considering choosing sites that differed dramatically in a geographic sense. The main differences between the four sites in our experiment were water availability (Supplemental Table S1). The sum of daily rainfall and the degree days collected during the safflower growing season in 2017 and 2018 indicated that Sites 1 and 2 experienced a wet and warm spring followed by a dry and mild summer. Site 1 had a full soil water profile with irrigation before sowing, so we consider Site 1 to have good water conditions. Site 2 experienced less rain after November, with high biomass accumulation during the spring and less soil water available in the late spring, therefore this site experienced drought stress starting in November, which was the flowering stage. Site 4 had more rain in total than Site 3; however, a dry and cold spring followed by a dry and hot summer indicated that both sites were under drought stress from the early vegetative stage until harvest. The lower grain yield (YP) with a shorter flowering period (FP) at Site 2 than at Site 1 indicated that water availability differed from the two sites. Drought stress at the flowering stage could heavily impact safflower grain yield. Studies with wheat showed that grain yield is most sensitive to stresses during flowering time as grain number is determined just prior to and at flowering (Fischer, 2009; Flohr, Hunt, Kirkegaard, & Evans, 2017). However, safflower differs from wheat in that it is indeterminant, and we suggested that the YP loss in Site 2 could be due to the decrease of the seed numbers per plant, possibly by fewer capsules forming and/or through less seed set per capsule because 500 seed weight did not change dramatically between Site 1 and Site 2 (Figure 1). Our assumption was supported by a study with 160 safflower accessions, which revealed a high correlation (r = 0.89) between seed number per plant and grain yield (Yared & Misteru, 2016). Safflower was known as a drought‐tolerant crop, but drought during early safflower growth stages could still have a significant impact on grain yield (Yau, 2007). In our experiment, low plant height (PH) and low grain yield at Sites 3 and 4 demonstrated that a lack of water in Spring could be a challenge to grow safflower in the region when rotating with other crops. The impact of rainfall and temperature conditions on phenology caused Sites 3 and 4 to mature early, presumably due to hot, dry conditions putting plants under severe terminal water stress.

The water availability differences across sites impacted not only the performance of safflower accessions but also the heritability estimation for YP and its related traits. Both estimated *H* ^2^ and *h* ^2^ were reduced in Sites 3 and 4 (stressed environments) for PH and YP. YP showed the lowest average *H* ^2,^ and *h* ^2^ among all the traits studied. Low *h* ^2^ for safflower seed yield per plant at 0.09 has been previously reported (H Pahlavani, Saeidi, & F Mirlohi, 2007). In other crops, the low broad‐sense heritability and low genomic heritability for grain yield have also been observed (Habib, Amine, Sophie, Richard, & Rod, 2016; Thorwarth et al., 2017; Velazco et al., 2019). High *h* ^2^, at 0.79 for safflower plant height was observed from a crossing population, and the genetic study indicated that additive genes were the main genetic component controlling inheritance of plant height (Golkar, Arzani, & Rezaei, 2012; Kotecha, 1979). In our study, we observed a low level of g × E interaction and moderate *h* ^2^ for PH, suggesting that additive genetic effects accounted for the moderate part of the total PH variance. With high *h* ^2^_ge_ observed across sites, it indicated that safflower PH observed in one environment could be able to moderately predict PH in another environment. A study on rice plant height showed similar genomic heritability (0.95), and the prediction accuracy was above 0.70 (Isidro et al., 2015). 500 seed weight (SW) estimates in our study were consistent with previous parent‐offspring regression studies in safflower, where *H* ^2^ ranged from 0.66 to 0.86 (Kotecha, 1981), and *h* ^2^ was 0.55 for SW (Pahlavani, Saeidi, & Mirlohi, 2012). Due to the lack of g × E interaction in SW, a much higher *h* ^2^_ge_ indicated much higher repeatability for SW across environments. Limited environmental influence on SW had been observed in a pea diversity panel (Burstin et al., 2015). Variance components for phenology traits varied across sites. Traditionally, safflower was grown for its flowers that are used in dyes and medicine, also as fresh‐cut and dried flowers in different countries. Studies with DF and DM in safflower found that non‐additive genetic effects were involved in the genetic control of those two traits (Golkar et al., 2012; Kotecha, 1979). In our study, we observed a high phenotypic correlation between those two traits. With a moderate *h* ^2^, and low g × E interaction for DF and DM, suggested that additive genetic variance effects accounted for more than half of the phenotypic variance. Studies with soybean showed a similar result for days of maturity (Jarquín et al., 2014). Supplemental Figure S2 showed the distribution of the phenology traits in the four environments, which indicated that efficient selection for earliness or lateness in safflower foster adaptation to local environments is promising. Heritability is the key parameter in determining the efficiency of genomic prediction traits (Daetwyler, Villanueva, & Woolliams, 2008). The genomic parameters estimated in the study will be useful for the future implementation of genomic selection in safflower breeding.

Genomic prediction applied in the genebank germplasm evaluation process could help to accelerate the mining of genetic diversity from genebanks. In our study, a diverse safflower genebank germplasm collection, which included both landraces collected from throughout the world and elite cultivars and breeding lines, was used as a training population for the genomic prediction. Moderate to high prediction accuracy (PA) was achieved across the four sites for safflower grain yield and its related traits using a GBLUP model in our study. Similar or higher PA were achieved for grain yield‐related traits in other oilseed crops (Reif, Zhao, Würschum, Gowda, & Hahn, 2013; Würschum, Abel, & Zhao, 2014). We combined the four sites and included the interaction between the additive genetic effects and sites (g × E interaction) in our g × E GBLUP model. PA only improved when accessions were present at some sites (CV 2 scenario), consistent with a previous study (Burgueño, de los Campos, Weigel, & Crossa, 2012). A study in forest trees showed that individuals could be predicted with high PA across environments in traits with low g × E interactions (Li, Suontama, Burdon, & Dungey, 2017). The relatively low g × E interaction, and moderate to high *h* ^2^_ge_ for all traits investigated in this study, indicated that the *h* ^2^ had moderate to high repeatability across sites. With combined accessions information across sites, a more accurate PA could be achieved in the CV2 scenario. For example, no g × E interaction was observed for SW, and its PA was 0.94 in the CV2 scenario. Our results indicated that applying the g × E GBLUP model could potentially lower the cost of field testing for germplasm evaluation as not all lines need to be grown at all sites. In addition, the CV1 scenario showed comparable PA to the single site GBLUP, showing that the prediction of completely untested samples was possible. Our study has demonstrated that genomic prediction is feasible in safflower Genebank accessions evaluation.

Genotyping by sequencing (GBS) is a cost‐effective genotyping method that is widely adopted in genomic studies in different crops. A variety of SNP genotype missing rates and MAF thresholds have been applied in different genomic selection (GS) studies with GBS datasets (Haile et al., 2018; Sverrisdottir et al., 2017). Studies in simulated and empirical datasets have shown that prediction accuracies plateau at a certain marker density, depending on population LD and the trait genetic architecture (Abed, Perez‐Rodriguez, Crossa, & Belzile, 2018; Meuwissen, 2009). In our study, PA for YP was unaffected by missing genotype data rates from 10% to 80%, indicating that the lower number of SNPs from a stringent 10% SNP missing rate were sufficient to achieving accurate genomic prediction for YP. However, different SNP missing rates affected *h* ^2^ estimates (Figure 3). The susceptibility of *h* ^2^ estimates to GBS data quality thresholds has been previously reported (Ashraf et al., 2016; Dodds et al., 2015). The accurate estimation of relatedness between individuals, represented by **G,** is important to decompose the total variance, which would affect heritability estimation (Goddard, Hayes, & Meuwissen, 2011). In our study, reduced LD, increased diagonals of **G,** and decreased off‐diagonals of **G** with increased SNP missing rate indicated that the population appears more diverse, and the within‐individual diversity decreased. We propose that the additional SNP gained from relaxing the SNP missing rate either only provided information that is already captured or that the higher missing rate deteriorated the quality and advantage of the additional SNPs. The apparent trend in decreasing relatedness changed the ratio of between‐to‐within line variance, causing heritability estimates to rise (Figure 3). Changing the MAF threshold did not impact PA, which is consistent with a previous study (Zhang, Yin, Wang, Yuan, & Liu, 2019). By using the relative levels of *H* ^2^ to *h* ^2^ as our main criterion, we concluded that an SNP missing rate < 30% and MAF > 0.05 was a robust GBS data quality control combination for GP in our study.

## 5 CONCLUSION

Compared to major crops, safflower breeding has received less research attention due to its reduced prominence in agricultural systems. In our study, the safflower genebank collection was clustered into seven groups. Moderate PA was achieved for a range of traits using both GBLUP and g × E GBLUP models, demonstrating the potential for GP in the genetic evaluation of safflower. Genomic heritability was affected by the quality control filters applied to the GBS genotype data, while PA was more robust. The diverse genetic background of our germplasm provides a vital resource for safflower germplasm improvement and a valuable training set for genomic prediction models. The findings of the study will facilitate germplasm evaluation and safflower breeding via GP.

## AUTHOR CONTRIBUTIONS STATEMENT

HZ, MH and HD conceived and designed the experiment. SL, JP and ML performed and supervised the phenotyping and genotyping and reviewed the manuscript. YL, MH and HD supervised the statistical analysis and reviewed the manuscript. HZ performed the data analysis and interpreted the results. HZ and HD wrote the manuscript.

## CONFLICT OF INTEREST STATEMENT

The authors declare that there is no conflict of interest

## Supporting information

SUPPORTING MATERIAL

SUPPORTING MATERIAL

## Data Availability

Data available upon request for research purposes.
